# 3D lattice distortions and defect structures in ion-implanted nano-crystals

**DOI:** 10.1038/srep45993

**Published:** 2017-04-06

**Authors:** Felix Hofmann, Edmund Tarleton, Ross J. Harder, Nicholas W. Phillips, Pui-Wai Ma, Jesse N. Clark, Ian K. Robinson, Brian Abbey, Wenjun Liu, Christian E. Beck

**Affiliations:** 1 Department of Engineering Science, University of Oxford, Parks Road, Oxford, OX1 3PJ, UK; 2 Department of Materials, University of Oxford, Parks Road, Oxford, OX1 3PH, UK; 3 Advanced Photon Source, Argonne National Laboratory, Argonne, Illinois 60439, USA; 4 ARC Centre for Advanced Molecular Imaging, Department of Chemistry and Physics, La Trobe Institute for Molecular Science, La Trobe University, Victoria 3086, Australia; 5 CSIRO Manufacturing Flagship, Parkville 3052, Australia; 6 Culham Centre for Fusion Energy, Culham Science Centre, Abingdon, Oxfordshire, OX14 3DB, UK; 7 Stanford PULSE Institute, SLAC National Accelerator Laboratory, Menlo Park, CA 94025, USA; 8 Condensed Matter Physics and Materials Department, Brookhaven National Laboratory, 734 Brookhaven Avenue, Upton, NY, 11973, USA

## Abstract

Focussed Ion Beam (FIB) milling is a mainstay of nano-scale machining. By manipulating a tightly focussed beam of energetic ions, often gallium (Ga^+^), FIB can sculpt nanostructures via localised sputtering. This ability to cut solid matter on the nano-scale revolutionised sample preparation across the life, earth and materials sciences. Despite its widespread usage, detailed understanding of the FIB-induced structural damage, intrinsic to the technique, remains elusive. Here we examine the defects caused by FIB in initially pristine objects. Using Bragg Coherent X-ray Diffraction Imaging (BCDI), we are able to spatially-resolve the full lattice strain tensor in FIB-milled gold nano-crystals. We find that every use of FIB causes large lattice distortions. Even very low ion doses, typical of FIB imaging and previously thought negligible, have a dramatic effect. Our results are consistent with a damage microstructure dominated by vacancies, highlighting the importance of free-surfaces in determining which defects are retained. At larger ion fluences, used during FIB-milling, we observe an extended dislocation network that causes stresses far beyond the bulk tensile strength of gold. These observations provide new fundamental insight into the nature of the damage created and the defects that lead to a surprisingly inhomogeneous morphology.

The ability of FIB to shape materials at the nano-scale has made it central to microchip prototyping^1^, 3D material analysis^2^,^3^, targeted electron microscopy sample extraction^4^,^5^ and the nanotechnology behind size-dependent material properties^6^,^7^, to name but a few examples. It is tempting to assume FIB milling to be atomically perfect, only removing surface atoms. This is unfortunately not the case: sufficiently energetic incident ions will displace target atoms from their equilibrium lattice positions, causing collision cascades and structural damage^8^. Predicting this ion-implantation damage and its effect on material properties is not straightforward^9^,^10^. The effects of ion-implantation generally remain poorly understood. Yet they have important consequences. For example, FIB-milled nano-structures have been used extensively to investigate the size-dependence of material properties, leading to the “smaller is stronger” paradigm^6^,^7^. However, several studies suggest that FIB-induced defects may themselves be a major contributor to the observed scale-dependence of material strength^11^,^12^.

The damage produced by FIB-milling ranges from amorphization^13^ to the generation of lattice defects^12^ and the formation of intermetallic phases^14^. To examine its effect on material properties, detailed measurements of the lattice strains that govern defect interactions are essential. Previously, FIB-induced strains were inferred by considering the deflection of FIB-milled cantilevers^15^. However, such coarse measurements cannot capture the rich detail of heterogeneous defect distributions that determine material behaviour.

To investigate in detail the complex changes brought about by FIB milling we concentrate on the non-destructive, three-dimensional nano-scale measurement of FIB-milling-induced lattice strains in initially pristine objects. This is distinct from numerous previous studies where FIB milling was used to relieve pre-existing residual strains^16^,^17^,^18^. Rather our experiments quantify the strains directly caused by the defects introduced by FIB-milling. Gold was chosen as a model system since near-perfect nano-crystals can be reliably grown^19^. Our experiments use non-destructive Bragg Coherent X-ray Diffraction Imaging (BCDI)^20^, where a 3D coherent X-ray diffraction pattern (CXDP), i.e. an oversampled 3D reciprocal space map, is collected from a coherently illuminated single crystal (Fig. 1(A))^21^. The CXDP corresponds to the intensity of the 3D Fourier transform of the electron density in the crystal. By recovering the phase of the CXDP, the real space, complex-valued, electron density can be reconstructed^20^. Its amplitude provides information about electron density, *ρ*(**r**), i.e. the shape of the crystal. Its phase, *ψ*(**r**), is linked to displacements, **u**(**r**), of atoms from their ideal lattice positions by *ψ*(**r**) = **q.u**(**r**), where **q** is the scattering vector of the CXDP. By combining at least three CXDPs with linearly independent **q** vectors, **u**(**r**) can be recovered^22^, and the full lattice strain tensor, **ε**(**r**), determined by differentiating **u**(**r**). Thus BCDI allows the 3D nano-scale measurement of both crystal morphology and the full lattice strain tensor.

## Results

### Lattice Distortions due to FIB Imaging

**Figure 1:**
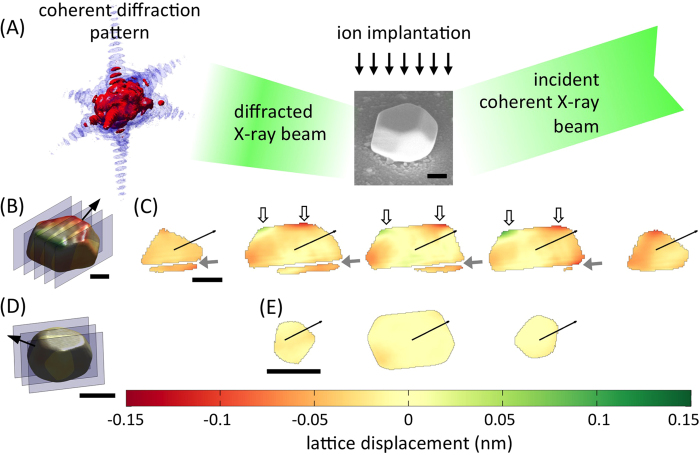
Lattice displacements due to FIB imaging. (**A**) Schematic of the experimental configuration showing the illuminating and scattered beams, an SEM micrograph of gold crystal A and 3D rendering of the (1-11) CXDP from this crystal. Low dose FIB-imaging (4.2 × 10^4^ ions/μm^2^) was applied to the top crystal surface at normal incidence. (**B**) Reconstruction of crystal A based on the (1-11) CXDP and coloured by lattice displacement. (**C**) Cross-sections through the reconstructed displacement field of crystal A ((**B**) shows locations of sections). Missing intensity due to a twin domain (grey arrows) and large lattice displacements near the implanted top surface (white arrows) are visible. (**D**) Reconstruction of an unimplanted gold nano-crystal based on a {111} CXDP, and colour-coded by lattice displacement. (**E**) Cross-sections through the displacement field of the unimplanted crystal ((**D**) shows locations of sections). Lattice displacements in (**B**–**E**) are in the scattering vector direction (thin black arrow). Scale bars are 300 nm in length.

First we consider the effect of FIB-imaging. Before X-ray measurements nano-crystal A was exposed to a Ga^+^ dose just sufficient to image the sample (30 keV, 50 pA, 4.2 × 10^4^ ions/μm^2^). The crystal morphology (Fig. 1(B)), reconstructed with a spatial resolution of ~38 nm based on the (1-11) CXDP, is in excellent agreement with scanning electron micrographs (Fig. 1(A) and SI Fig. S1). The 3D field of lattice displacements in the [1-11] crystal direction (Fig. 1(B,C)) shows large displacements near the top, implanted, surface of the crystal. By comparison, the lattice displacements measured in an unimplanted, as-grown crystal, reconstructed with ~12 nm spatial resolution, are small (Fig. 1(D,E)). The unimplanted crystal only shows slight increases in lattice displacement at its vertices due to surface energy effects^23^. Displacements in the vicinity of the crystal-substrate interface are small, indicating negligible substrate-induced strains in the crystals in contrast to other, previously studied material systems^21^. This demonstrates that the large displacements in crystal A are caused by the Ga^+^ bombardment.

**Figure 2:**
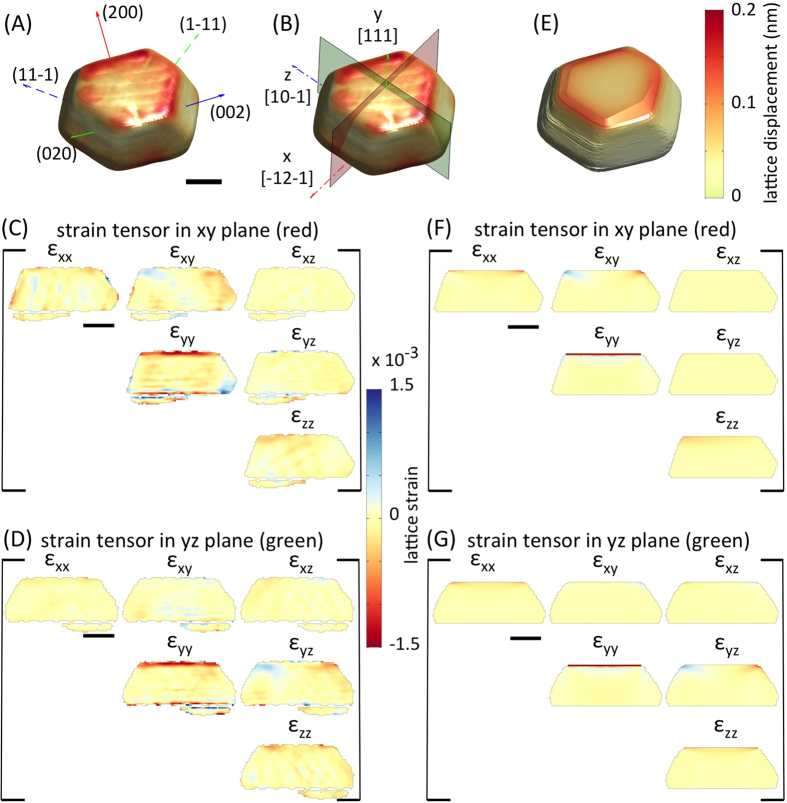
Full 3D lattice strain tensor after FIB imaging (crystal A). (**A**) 3D rendering of crystal A coloured by lattice displacement magnitude. Superimposed are the **q** vectors of the five measured crystal reflections. (**B**) Coordinate system used for plotting lattice strains and sections on which strains are plotted. The x, y and z axes correspond to [-12-1], [111] and [10-1] crystal directions respectively. (**C**) and (**D**) Maps of the six independent lattice strain tensor components on the xy section (red plane in (**B**)) and yz section (green plane in (**B**)) through crystal A respectively. (**E**) Finite element model of crystal A showing the predicted displacement field magnitude. Calculated lattice strain components are plotted on the same xy (**F**) and yz (**G**) planes as the experimental data. Scale bars are 300 nm in length.

To further explore these FIB-induced lattice distortions, CXDPs from five reflections were used to reconstruct the full 3D-resolved lattice strain tensor, **ε**(**r**), inside crystal A (Fig. 2). The six independent components of **ε**(**r**) are shown on virtual xy and yz sections through crystal A (Fig. 2). ε_yy_(**r**) is large and negative within ~30 nm of the implanted top surface, indicating a lattice contraction due to Ga^+^ implantation. The ε_xy_(**r**) (Fig. 2(C)) and ε_yz_(**r**) (Fig. 2(D)) shear components show more subtle strain features.

These strains can be understood by direct comparison with numerical calculations. Using the measured 3D morphology, an anisotropically elastic^24^ finite element (FE) model of crystal A was constructed (Fig. 2(E), SI Fig. S2). Simulations using the Stopping Range of Ions in Matter (SRIM) code^25^ predict a ~20 nm thick damage layer. The calculated implantation profiles are shown in SI Fig. S3. Accordingly a constant volumetric Eigenstrain, ε_v_, was imposed within a 20 nm thick surface layer in the FE model. ε_v_ = −3.15 × 10^−3^ provides a good match to the experimentally measured lattice displacements. There is striking agreement between calculated strains (Fig. 2(F,G)) and measured strains (Fig. 2(C,D)). Not only are the ε_yy_(**r**), ε_xy_(**r**) and ε_yz_(**r**) components well matched, but finer features in the other strain components also agree. This highlights that even very low Ga^+^ fluences lead to substantial lattice distortions, and demonstrates the unique capability of BCDI for detailed, 3D-resolved nano-scale strain analysis. The magnitude of the FIB-induced eigenstrain is similar to previous observations in silicon inferred from the distortion of FIB-milled cantilevers^15^.

The volumetric lattice strain due to defects is given by 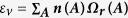, where 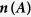 and 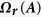 respectively are number density and relaxation volume of defect type *A*^10^. Using density functional theory (DFT) calculations we found that for a gold monovacancy Ω_r_(V) = −0.38, whilst all self-interstitial atom (SIA) configurations (100 dumbbell, octahedral site, 110 crowdion and 110 dumbbell, SI Fig. 4) have Ω_r_(SIA) = 2.0. Hence collision damage in the bulk, involving equal numbers of SIAs and vacancies, will cause a lattice swelling. The observed lattice contraction is surprising, particularly since the relaxation volume of substitution gallium in gold is small and positive (SI Table S1). Thus the lattice contraction we measure must indicate an excess of vacancies. This can be explained by considering the proximity of the crystal surface: vacancies and vacancy clusters with high migration energy (*E*_*m*_ ≥ 0.71 eV)^26^,^27^,^28^ are retained, while SIAs, which are mobile even at temperatures of a few K^28^, escape to the free surface. ε_v_ and Ω_r_(V) allow a lower bound estimate of ~230 retained vacancies/(Ga^+^), while SRIM calculations provide an upper bound of ~400 vacancies/(Ga^+^). Based on the implanted Ga^+^ dose and ε_v_, these bounds correspond to defect concentrations of ~4.9 × 10^26^ vacancies/m^3^ (lower bound) and ~8.5 × 10^26^ vacancies/m^3^ + 6.79 × 10^25^ SIAs/m^3^ (upper bound) respectively. Thus our measurements allow quantitative insight into the nature of the damage formed, even at very low ion fluences.

### Higher Dose FIB Milling

**Figure 3:**
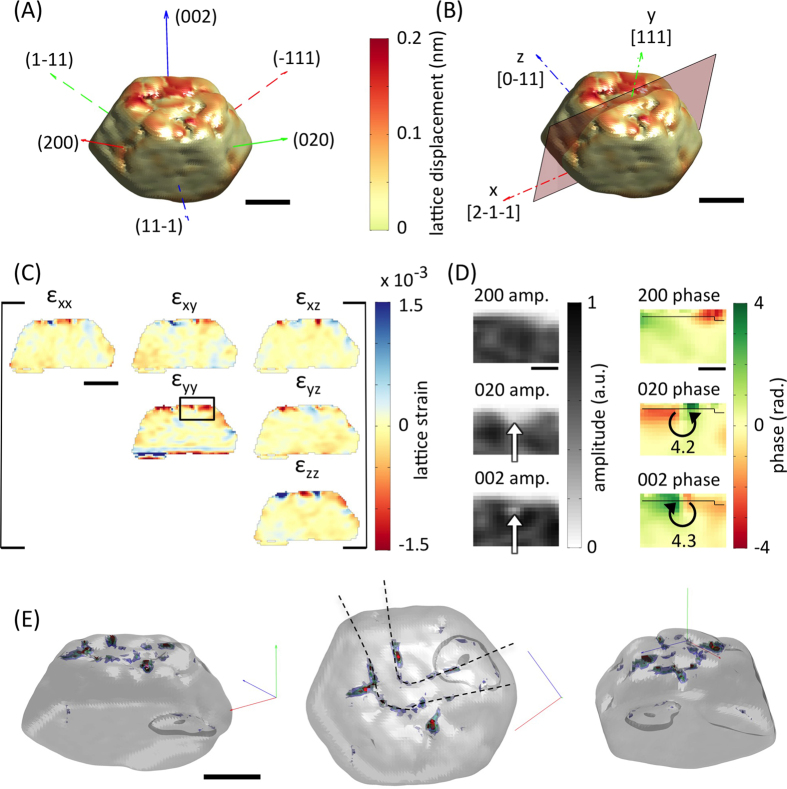
Displacements, strains and stresses after FIB milling (crystal C). (**A**) 3D rendering of crystal C reconstruction coloured by lattice displacement magnitude. **q** vectors of the six measured reflections are superimposed. (**B**) Crystal coordinates and section on which the lattice strains are plotted. The x, y and z axes correspond to [-12-1], [111] and [10-1] crystal directions respectively. (**C**) Maps of the six independent lattice strain tensor components plotted on the xy section shown in (B). (**D**) Magnified view of amplitudes and phases of the complex electron density reconstructed from {200} reflections. The region corresponds to that marked by a black box in (**C**) and is centred on a defect. Areas of reduced amplitude (white arrows) and phase jumps (in radians, circular arrows) are visible in the (020) and (002) reflections. (**E**) Semi-transparent rendering of the outer crystal shape. Superimposed are iso-surfaces of von Mises stress (300 MPa (blue), 400 MPa (green), 500 MPa (red)). Three different viewpoints are shown. In the middle view dashed black lines have been superimposed as a guide to the eye to illustrate the arrangement of defects in lines. Scale bars correspond to 300 nm in (**A**–**C**) and (**E**), and 100 nm in (**D**).

At higher Ga doses a distinctly different behaviour is observed. Nano-crystals B and C were exposed to fluences of 1.3 × 10^7^ ions/μm^2^ and 1.5 × 10^8^ ions/μm^2^ respectively, causing the removal of ~3 nm and ~40 nm thick surface layers by sputtering, as predicted by SRIM. Lattice displacements and strains in both crystals were reconstructed using six crystal reflections (SI Fig. S5 and Fig. 3 respectively). The spatial resolutions of these reconstructions are ~45 nm and ~47 nm respectively. Even for these highly damaged crystals agreement of the reconstructed morphology and SEM micrographs is excellent (SI Fig. S1).

The lattice displacement magnitude in crystal C (Fig. 3(A)) shows abrupt variations, in contrast to the gradual changes in crystal A (Fig. 2(A)). The ε_yy_(**r**) strain (Fig. 3(C)) is no longer uniform and negative in the implanted layer, but contains compressive and tensile regions. Similar variations are present in the other strain components.

The nature of the underlying crystallographic defects can be explored by considering the amplitudes and phases of the complex electron density reconstructed from different crystal reflections. Fig. 3(D) shows the amplitudes and phases of the (200), (020) and (002) reflections for the area marked by a black rectangle in Fig. 3(C). The (020) and (002) reflection phases both show a phase jump of ~4.2 radians, while the (200) reflection phase shows no discontinuity. The structure of the phase jumps suggests dislocations as the underlying defects^29^,^30^,^31^. The magnitude of the phase jump, Δ*ψ*_hkl_, anticipated due to a dislocation with Burgers vector **b** observed in a given *hkl* reflection is Δ*ψ*_hkl_ = **b.q**_hkl_. The scattering vectors associated with the (200), (020) and (002) reflections respectively are: **q**_200_ = (2π/*a*) [200], **q**_020_ = (2π/*a*) [020] and **q**_002_ = (2π/*a*) [002], where *a* is the lattice parameter. This suggests that the defect in Fig. 3(D) is a dislocation with Burgers vector (*a*/3)[01-1]. Such a so-called stair-rod dislocation can be formed through the interaction of two Shockley partial dislocations^32^. For example in the present case the energetically favourable reaction (*a*/6)[21-1] + (*a*/6)[−21-1] **→** (*a*/3) [01-1] would produce a sessile dislocation with the observed Burgers vector.

The amplitude maps associated with reflections where phase jumps are observed show a local reduction in intensity at the defect position. Both (020) and (002) reflections show this reduction (white arrows in Fig. 3(D)), while no such feature is observed in the (200) reflection. This agrees with BCDI observations of “pipes of missing intensity” at dislocation cores^30^. Indeed throughout the crystal, several further defects consistent with (*a*/3) <110> stair-rod dislocations^32^ can be identified (Supplementary Note 1.1, SI Fig. S6).

The ordering of larger defects in crystal C can be visualized by computing the von Mises stress^33^. Figure 3(E) shows von Mises stresses >500 MPa, greatly exceeding the yield strength of bulk gold (55–200 MPa)^34^. The arrangement of defects in lines is unexpected and differs from TEM observations of uniformly distributed (<100 nm) dislocation loops in FIB-milled copper^12^. The fact that we only observe sessile stair-rod dislocations is surprising. It suggests substantial evolution of the damage microstructure after the initial collision cascade with mobile dislocations escaping to the free surface and only sessile dislocations remaining.

**Figure 4:**
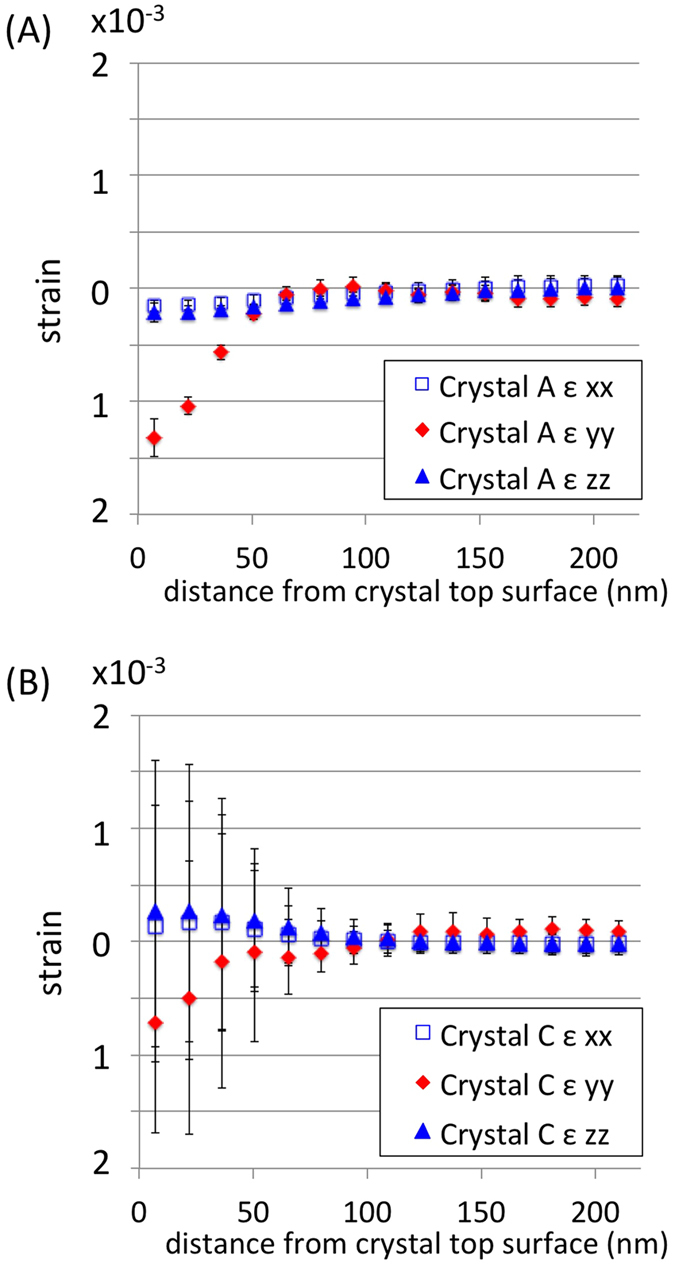
Strain variation with distance from the ion-implanted surface. (**A**) Line profiles of normal strain, ε_yy_, and in-plane strains, ε_xx_ and ε_zz_, plotted as a function of distance from the gallium-implanted surface of crystal A. (**B**) Line profiles of ε_xx_, ε_yy_ and ε_zz_ plotted as a function of distance from the gallium-implanted surface of crystal C. Markers show the average strain value at each depth. The error bars capture the standard deviation of ε_xx_, ε_yy_ and ε_zz_ at each specific depth, considering variation over a 0.3 × 0.3 μm^2^ area. The x, y and z directions follow the conventions shown in Figs 2(B) and 3(B) for crystals A and C respectively.

It is interesting to consider the effect of these defects on the average strains induced by FIB milling. Figure 4 shows profiles of ε_xx,_ ε_yy,_ and ε_zz_ for crystals A and C plotted as a function of depth from the implanted surface. ε_yy_ in the implanted layer of crystal A is approximately twice as large as in crystal C, but has much less variation. This suggests that at higher Ga^+^ fluences larger defects, as well as the clustering of point defects^10^, act to relieve implantation-induced strains by localizing lattice distortion.

## Discussion

**Figure 5:**
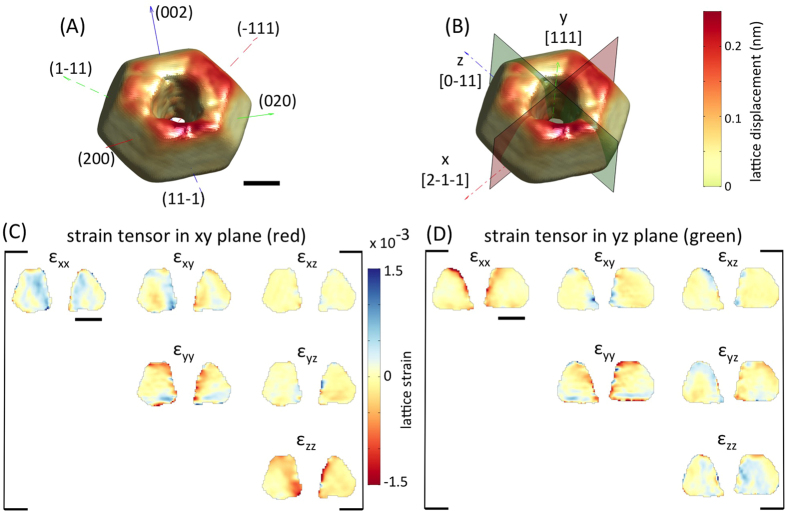
Strains induced by extensive FIB milling (crystal D). (**A**) 3D rendering of crystal D coloured according to the measured lattice displacement magnitude. Superimposed are the **q** vectors of the six reflections measured from this crystal. (**B**) Crystal coordinates and sections on which strains are plotted. The x, y and z axes correspond to [2-1-1], [111] and [0-11] crystal directions respectively. (**C**) and (**D**) show the six components of the experimentally measured strain tensor plotted on xy and yz sections through the crystal (shown in (**B**) coloured red and green respectively). Scale bars are 300 nm in length.

Our findings show that every use of FIB to image or shape material causes large lattice distortions. Fundamental insight into the underlying damage mechanisms can be gained by combining coherent X-ray measurements with numerical calculations. Surprisingly, FIB-induced lattice strains are not confined to the ion-damaged layer, but can extend far into the material bulk, as visible in crystal C. This is further highlighted by measurements of crystal D into which a central hole was FIB-machined, and which exhibits large strains even far from the ion-damaged surfaces (Fig. 5). These extensive strains may explain the dramatic changes in mechanical properties caused by FIB milling^11^,^35^,^36^.

Our observations emphasise the need to actively consider the defects produced during FIB-based nano-fabrication. For example FIB-assisted deposition of a protective layer (usually Pt, W or C) is often used to prevent sample surface damage^4^. Our results suggest that even with such a protective layer substantial strains and damage should be expected. The reason is Ga^+^ damage formed during the initial stages of layer deposition before the layer thickness exceeds the Ga^+^ range. In the present gold crystals, FIB deposition of a Pt layer^37^,^38^,^39^ would cause defects similar to those in crystals A and B. E-beam-assisted deposition during the initial stages of layer growth could avoid this problem.

To realise the full potential of FIB, new strategies for controlling and minimising FIB damage must be developed. Current approaches include using a final low current milling step^40^,^41^,^42^, low energy ion milling^43^,^44^ or flash polishing^45^ in order to “clean” FIB-damaged surfaces. The techniques presented here enable 3D measurements of the complex strain fields caused by FIB-milling and would as such allow a detailed assessment of the effectiveness of these approaches. Conversely the ability of FIB to introduce large lattice strains with high spatial specificity presents an exciting opportunity for modifying material behaviour through strain engineering at the nano-scale.

## Methods

### Sample Manufacture

Gold crystals were prepared by dewetting a 20 nm thick gold layer, thermally evaporated onto a silicon substrate with a 2 nm titanium adhesion layer. The resulting crystals range from ≈100 nm to a few μm in size and show facets corresponding to {111} and {100} crystal planes (Fig. 1(A)). No FIB-milling was carried out in the vicinity of the unimplanted reference crystal. FIB-milling of crystals A, B and C was carried out at normal incidence, using a 30 keV, 50 pA gallium ion beam and fluences of 4.2 × 10^4^ ions/μm^2^, 1.3 × 10^7^ ions/μm^2^ and 1.5 × 10^8^ ions/μm^2^ respectively. Crystal D was exposed to a fluence of 4.2 × 10^4^ ions/μm^2^ and a central, nominally 200 nm diameter, region to a fluence of 2.5 × 10^9^ ions/μm^2^. To allow reliable measurement of multiple reflections from crystals A, B, C and D, FIB was used to remove any other gold crystals within a 20 μm radius. Scanning electron micrographs of crystals A, B, C and D are shown in the SI Fig. S1. X-ray diffraction measurements were carried out 16 to 20 days after sample manufacture.

### Ion Implantation Calculations

Ion implantation calculations used the “monolayer collision - surface sputtering” model in the Stopping Range of Ions in Matter (SRIM) code^25^. For the gold target a displacement energy of 44 eV, binding energy of 3 eV and surface energy of 3.8 eV were used^46^. Gallium ions were implanted at normal incidence with an energy of 30 keV, gathering statistics over 10^5^ ions. Each ion was estimated to cause on average ~430 target displacements, of which ~30 were replacement collisions. The calculated sputtering rate was ~15.5 gold atoms per gallium ion. For crystal A the amount of material removed by sputtering was negligible. For crystals B and C, an estimated layer of thickness ~3 nm and ~40 nm respectively was removed. Custom MATLAB scripts were used to capture the receding surface effect due to sputtering. The calculated displacement damage and gallium concentration profiles for crystals A, B, C and D, plotted as a function of depth, are shown in the SI Fig. S3.

### Experimental Measurements

Synchrotron X-ray micro-beam Laue diffraction at beamline 34-ID-E at the Advanced Photon Source (APS), Argonne National Lab, USA was used to determine the lattice orientations of gold crystals. This served to pre-align crystals for coherent X-ray diffraction measurements at beamline 34-ID-C at the APS. Measurements on the unimplanted reference crystal used an X-ray energy of 9.25 keV, while diffraction patterns from crystals A, B, C and D were collected at 10.2 keV. The X-ray beam was focussed to a size of 1.4 × 2.1 μm^2^ (h × v) using KB mirrors. Placing the sample in the KB back-focal plane, within the central maximum of the focus, provides the planar wave front required for BCDI. Diffraction patterns were recorded on a Medipix2 area detector with a 256 × 256 pixel matrix and a pixel size of 55 × 55 μm^2^. For crystals A, B, C and D the detector was positioned 1.85 m from the sample and 3D coherent X-ray diffraction patterns (CXDP) were recorded by rotating the crystal through an angular range of 0.4° and recording an image every 0.0025° with 1 s exposure time. For the unimplanted reference crystal a sample-to-detector distance of 0.635 m was used and CXDPs were recorded by rotating through an angular range of 1.5° in 0.01° steps with 0.5 s exposure time. The sample to detector distances were chosen by starting at the distance required for the measurement of an oversampled diffraction pattern and then moving the detector further back until the diffraction pattern filled the detector matrix. To optimize the signal to noise of the CXDPs, multiple repeated scans of each reflection were performed. Repeated scans were then aligned to maximize their cross-correlation coefficient, and scans with a cross-correlation coefficient greater than 0.99 were summed to produce the CXDP for a specific reflection. For each crystal CXDPs from the following reflections were collected (the number of repeat scans that were averaged is noted in [] brackets): unimplanted reference: {111} [30]; crystal A: (1-11) [18], (11-1) [24], (200) [23], (020) [26], (002) [27]; crystal B: (-111) [16], (1-11) [9], (11-1) [14], (200) [11], (020) [16], (002) [17]; crystal C: (-111) [28], (1-11) [14], (11-1) [27], (200) [26], (020) [22], (002) [18]; and crystal D: (-111) [12], (1-11) [16], (11-1) [17], (200) [9], (020) [12], (002) [14]. Unfortunately the (-111) reflection of crystal A was physically inaccessible. Examples of the coherent diffraction patterns recorded from {111} reflections of all crystals are shown in SI Fig. S7.

### Phase Retrieval

The phase retrieval algorithm used to recover the real-space complex electron density is adapted from published work^30^. Each 3D CXDP pattern was treated independently, using a guided phase retrieval approach with 20 random starts and 5 generations. For each generation 330 phase retrieval iterations were performed using Error Reduction and Hybrid-Input-Output algorithms. Trials using larger numbers of iterations showed no significant further evolution of the solution. Partial coherence effects were accounted for^19^, and the normalised mutual coherence functions, recovered for all reflections, are consistent with an almost fully coherent illumination. After the fifth generation a sharpness metric was used to select the three best estimates, which were then averaged to return the reconstructed complex electron density. Finally all reconstructions were transformed into an orthogonal laboratory reference frame with isotropic real-space pixel spacing. Agreement between the reconstructed crystal morphologies and scanning electron micrographs is excellent (SI Fig. S1). The normalised cross correlation coefficients, found when comparing the sample shape recovered from BCDI with SEM images, are 0.97, 0.98, 0.97 and 0.97 respectively for crystals A, B, C and D, when considering sample shape projected onto the plane of the substrate. Spatial resolution of the reconstructions was determined by taking the derivative of line profiles of the crystal-air-interface and fitting these with a Gaussian. For each reconstruction six profiles (2 in each spatial direction) were measured and the mean resolution value recorded.

### 3D Reconstruction of Lattice Displacements, Strains and Stresses

To recover the 3D lattice displacement field, **u**(**r**), of a given crystal, any phase wraps in the complex electron densities reconstructed from multiple crystal reflections were unwrapped using the algorithm developed by Cusack *et al*.^47^. Next all reconstructions were rotated into the same sample coordinate frame. The phase of the electron density reconstructed from a particular *hkl* peak, *ψ*_*hkl*_(**r**), is linked to the scattering vector **q**_*hkl*_ and lattice displacement **u**(**r**) by *ψ*_*hkl*_(**r**) = **q**_*hkl*_**.u**(**r**). Thus each reconstruction provides a projection of **u**(**r**) along the corresponding **q**_*hkl*_. If 3 reflections with linearly independent **q**_*hkl*_ are measured, **u**(**r**) can be reconstructed. Here 5 (crystal A) or 6 (crystals B, C and D) reflections with non-collinear **q** vectors were measured from each crystal. Thus the system of equations is over determined, and a least squares fit was used to calculate **u**(**r**). The symmetric Cauchy strain tensor, **ε**(**r**), is found by differentiating **u**(**r**). The strain uncertainty of our measurements, estimated from line profiles of **ε**(**r**) extracted from crystal A (Fig. 4), is ~10^−4^. Stresses were computed from **ε**(**r**) using anisotropic elastic constants for gold^24^.

### Finite Element Calculations

Finite element simulations were performed in Abaqus 6.14, using the experimentally determined crystal morphology as a template for generation of the finite element mesh. Custom Matlab and Python scripts developed for this purpose are available upon request. A global seed size of 10 nm was used, based on mesh dependency studies that showed negligible improvements for finer mesh sizes. The resulting model for crystal A is shown in SI Fig. S2. Material properties were captured using anisotropic linear elastic constants for gold^24^. A uniform volumetric lattice strain, *ε*_*v*_, was imposed within a 20 nm thick layer at the top face of crystal A to represent the effect of ion-implantation damage. *ε*_*v*_* = *−3.15 × 10^−3^ provides a good match to the experimentally measured lattice displacement fields in crystal A. Displacements on the bottom surface of the crystal were fixed to capture the substrate effect.

### Density Functional Theory Calculations

*Ab initio* density functional theory (DFT) calculations were performed of a mono-vacancy and of four different self-interstitial defect configurations in fcc gold (100 dumbbell, octahedral site, 110 crowdion and 110 dumbbell). Calculations were carried out in the Vienna ab initio simulation package (VASP)^48^,^49^,^50^,^51^ using the revised-TPSS exchange functional^52^,^53^, and included spin-orbit coupling. Spin-orbit coupling accounts for the band splitting and shape modifications of the 5d bands^54^,^55^,^56^. A plane wave energy cutoff of 450 eV was used and the outermost s- and d-electrons were treated as valence electrons. All samples were relaxed to a stress free condition with residual forces smaller than 0.01 eV/Å. Formation energies and relaxation volumes were calculated by comparing the energies and volumes of a supercell containing each defect type with those of a perfect crystal supercell of similar size and using the same k-point mesh. Visualizations of the supercells used for these calculations are shown in SI Fig. S4.

The lattice constant and elastic constants were calculated using a 4 atom cubic unit cell. The equilibrium lattice constant is 4.075 Å in good agreement with experiments^57^. The elastic constants were calculated using a finite differences scheme. We obtained c_11_ = 210.55 GPa, c_12_ = 168.11 GPa and c_44_ = 49.96 GPa, which compare well to experimental values at 0 K^58^. The elastic constants were used to correct both formation energy and relaxation volume of isolated defects according to the method suggested by Varvenne *et al*.^59^, which considers the elastic interactions of defects, and image forces due to the finite supercell size and periodic boundary conditions. The formation energies and relaxation volumes are listed in SI Table S1. The relaxation volume of a substitution gallium atom in gold was also calculated.

### Data Availability

Diffraction data and selected computer codes used for data analysis and simulations can be obtained from the authors by contacting felix.hofmann@eng.ox.ac.uk.

## Additional Information

**How to cite this article:** Hofmann, F. *et al*. 3D lattice distortions and defect structures in ion-implanted nano-crystals. *Sci. Rep.* **7**, 45993; doi: 10.1038/srep45993 (2017).

**Publisher's note:** Springer Nature remains neutral with regard to jurisdictional claims in published maps and institutional affiliations.

## Supplementary Material

Supplementary Information

## References

[b1] Brousseau E. B., Dimov S. S. & Pham D. T. Some recent advances in multi-material micro- and nano-manufacturing. The International Journal of Advanced Manufacturing Technology 47, 161–180, doi: 10.1007/s00170-009-2214-5 (2009).

[b2] Inkson B. J., Mulvihill M. & Möbus G. 3D determination of grain shape in a FeAl-based nanocomposite by 3D FIB tomography. Scripta Materialia 45, 753–758, doi: 10.1016/S1359-6462(01)01090-9 (2001).

[b3] Lasagni F. . Three-dimensional characterization of ‘as-cast’ and solution-treated AlSi12(Sr) alloys by high-resolution FIB tomography. Acta Materialia 55, 3875–3882, doi: 10.1016/j.actamat.2007.03.004 (2007).

[b4] Mayer J., Giannuzzi L. A., Kamino T. & Michael J. TEM Sample Preparation and FIB-Induced Damage. MRS Bulletin 32, 400–407, doi: 10.1557/mrs2007.63 (2007).

[b5] Giannuzzi L. A. & Stevie F. A. A review of focused ion beam milling techniques for TEM specimen preparation. Micron 30, 197–204, doi: 10.1016/S0968-4328(99)00005-0 (1999).

[b6] Uchic M. D., Dimiduk D. M., Florando J. N. & Nix W. D. Sample Dimensions Influence Strength and Crystal Plasticity. Science 305, 986–989, doi: 10.1126/science.1098993 (2004). 15310897

[b7] Greer J. R. & De Hosson J. T. M. Plasticity in small-sized metallic systems: Intrinsic versus extrinsic size effect. Progress in Materials Science 56, 654–724, doi: 10.1016/j.pmatsci.2011.01.005 (2011).

[b8] Robinson M. T. & Torrens I. M. Computer simulation of atomic-displacement cascades in solids in the binary-collision approximation. Physical Review B 9, 5008–5024 (1974).

[b9] Yi X. . Direct observation of size scaling and elastic interaction between nano-scale defects in collision cascades. Europhysics Letters 110, 36001 (2015).

[b10] Hofmann F. . Lattice swelling and modulus change in a helium-implanted tungsten alloy: X-ray micro-diffraction, surface acoustic wave measurements, and multiscale modelling. Acta Materialia 89, 352–363, doi: 10.1016/j.actamat.2015.01.055 (2015).

[b11] Shim S., Bei H., Miller M. K., Pharr G. M. & George E. P. Effects of focused ion beam milling on the compressive behavior of directionally solidified micropillars and the nanoindentation response of an electropolished surface. Acta Materialia 57, 503–510, doi: 10.1016/j.actamat.2008.09.033 (2009).

[b12] Kiener D., Motz C., Rester M., Jenko M. & Dehm G. FIB damage of Cu and possible consequences for miniaturized mechanical tests. Materials Science and Engineering: A 459, 262–272, doi: 10.1016/j.msea.2007.01.046 (2007).

[b13] Giannuzzi L. A., Geurts R. & Ringnalda J. 2 keV Ga+ FIB Milling for Reducing Amorphous Damage in Silicon. Microscopy and Microanalysis 11, 828–829 (2005).

[b14] Yu J., Liu J., Zhang J. & Wu J. TEM investigation of FIB induced damages in preparation of metal material TEM specimens by FIB. Materials Letters 60, 206–209, doi: 10.1016/j.matlet.2005.08.018 (2006).

[b15] Salvati E., Sui T., Lunt A. J. G. & Korsunsky A. M. The effect of eigenstrain induced by ion beam damage on the apparent strain relief in FIB-DIC residual stress evaluation. Materials & Design 92, 649–658, doi: 10.1016/j.matdes.2015.12.015 (2016).

[b16] Sabaté N. . FIB-based technique for stress characterization on thin films for reliability purposes. Microelectronic Engineering 84, 1783–1787, doi: 10.1016/j.mee.2007.01.272 (2007).

[b17] Korsunsky A. M., Sebastiani M. & Bemporad E. Residual stress evaluation at the micrometer scale: Analysis of thin coatings by FIB milling and digital image correlation. Surface and Coatings Technology 205, 2393–2403, doi: 10.1016/j.surfcoat.2010.09.033 (2010).

[b18] Barabash R. I. . Mapping strain gradients in the FIB-structured InGaN/GaN multilayered films with 3D X-ray microbeam. Materials Science and Engineering: A 528, 52–57, doi: 10.1016/j.msea.2010.04.045 (2010).

[b19] Clark J. N., Huang X., Harder R. & Robinson I. K. High-resolution three-dimensional partially coherent diffraction imaging. Nature Communications 3, 993 (2012). 10.1038/ncomms1994 22871812

[b20] Robinson I. & Harder R. Coherent X-ray diffraction imaging of strain at the nanoscale. Nature Materials 8, 291–298 (2009). 19308088 10.1038/nmat2400

[b21] Pfeifer M. A., Williams G. J., Vartanyants I. A., Harder R. & Robinson I. K. Three-dimensional mapping of a deformation field inside a nanocrystal. Nature 442, 63–66 (2006). 16823449 10.1038/nature04867

[b22] Newton M. C., Leake S. J., Harder R. & Robinson I. K. Three-dimensional imaging of strain in a single ZnO nanorod. Nat Mater 9, 120–124 (2010). 20023632 10.1038/nmat2607

[b23] Robinson I. Nanoparticle Structure by Coherent X-ray Diffraction. Journal of the Physical Society of Japan 82, 021012, doi: 10.7566/JPSJ.82.021012 (2012).

[b24] Hiki Y. & Granato A. V. Anharmonicity in Noble Metals; Higher Order Elastic Constants. Physical Review 144, 411–419 (1966).

[b25] Ziegler J. F., Ziegler M. D. & Biersack J. P. SRIM–The stopping and range of ions in matter (2010). Nuclear Instruments and Methods in Physics Research, Section B: Beam Interactions with Materials and Atoms 268, 1818–1823 (2010).

[b26] Sonnenberg K. & Dedek U. Migration energy of single vacancies in gold. Radiation Effects 61, 175–178, doi: 10.1080/00337578208229930 (1982).

[b27] Ehrhart P., Carstanjen H. D., Fattah A. M. & Roberto J. B. Diffuse–scattering study of vacancies in quenched gold. Philosophical Magazine A 40, 843–858, doi: 10.1080/01418617908234878 (1979).

[b28] Atomic Defects in Metals Au (Springer-Verlag Berlin Heidelberg, 1991).

[b29] Takahashi Y. . Bragg x-ray ptychography of a silicon crystal: Visualization of the dislocation strain field and the production of a vortex beam. Physical Review B 87, 121201 (2013).

[b30] Clark J. N. . Three-dimensional imaging of dislocation propagation during crystal growth and dissolution. Nature Materials 14, 780–784, doi: 10.1038/nmat4320 (2015). 26030304 PMC4623157

[b31] Ulvestad A. . Topological defect dynamics in operando battery nanoparticles. Science 348, 1344–1347, doi: 10.1126/science.aaa1313 (2015). 26089511

[b32] Hirth J. P. & Lothe J. Theory of Dislocations. 2 edn (Wiley, 1982).

[b33] Hill R. The mathematical theory of plasticity(Clarendon Press, 1950).

[b34] Espinosa H. D., Prorok B. C. & Peng B. Plasticity size effects in free-standing submicron polycrystalline FCC films subjected to pure tension. Journal of the Mechanics and Physics of Solids 52, 667–689, doi: 10.1016/j.jmps.2003.07.001 (2004).

[b35] Bei H., Shim S., Miller M. K., Pharr G. M. & George E. P. Effects of focused ion beam milling on the nanomechanical behavior of a molybdenum-alloy single crystal. Applied Physics Letters 91, 111915–111913 (2007).

[b36] Lee S.-W., Mordehai D., Rabkin E. & Nix W. D. Effects of focused-ion-beam irradiation and prestraining on the mechanical properties of FCC Au microparticles on a sapphire substrate. Journal of Materials Research 26, 1653–1661, doi: 10.1557/jmr.2011.221 (2011).

[b37] Tao T., Wilkinson W. & Melngailis J. Focused ion beam induced deposition of platinum for repair processes. Journal of Vacuum Science & Technology B 9, 162–164, doi: 10.1116/1.585279 (1991).

[b38] Puretz J. & Swanson L. W. Focused ion beam deposition of Pt containing films. Journal of Vacuum Science & Technology B 10, 2695–2698, doi: 10.1116/1.586028 (1992).

[b39] Telari K. A. . Characterization of platinum films deposited by focused ion beam-assisted chemical vapor deposition. Journal of Vacuum Science & Technology B 20, 590–595, doi: 10.1116/1.1458958 (2002).

[b40] Gong J., Benjamin Britton T., Cuddihy M. A., Dunne F. P. E. & Wilkinson A. J. 〈a〉 Prismatic, 〈a〉 basal, and 〈c+a〉 slip strengths of commercially pure Zr by micro-cantilever tests. Acta Materialia 96, 249–257, doi: 10.1016/j.actamat.2015.06.020 (2015).

[b41] Liu L., Chen Z., Liu C., Wu Y. & An B. Micro-mechanical and fracture characteristics of Cu6Sn5 and Cu3Sn intermetallic compounds under micro-cantilever bending. Intermetallics 76, 10–17, doi: 10.1016/j.intermet.2016.06.004 (2016).

[b42] Žagar G., Pejchal V., Mueller M. G., Michelet L. & Mortensen A. Fracture toughness measurement in fused quartz using triangular chevron-notched micro-cantilevers. Scripta Materialia 112, 132–135, doi: 10.1016/j.scriptamat.2015.09.032 (2016).

[b43] McCaffrey J. P., Phaneuf M. W. & Madsen L. D. Surface damage formation during ion-beam thinning of samples for transmission electron microscopy. Ultramicroscopy 87, 97–104, doi: 10.1016/S0304-3991(00)00096-6 (2001). 11330503

[b44] Thompson K. . *In situ* site-specific specimen preparation for atom probe tomography. Ultramicroscopy 107, 131–139, doi: 10.1016/j.ultramic.2006.06.008 (2007). 16938398

[b45] Dawson K. & Tatlock G. J. Preparation of micro-foils for TEM/STEM analysis from metallic powders. Micron 74, 54–58, doi: 10.1016/j.micron.2015.04.006 (2015). 25967375

[b46] Jung P. Average atomic-displacement energies of cubic metals. Physical Review B 23, 664–670 (1981).

[b47] Cusack R. & Papadakis N. New Robust 3-D Phase Unwrapping Algorithms: Application to Magnetic Field Mapping and Undistorting Echoplanar Images. NeuroImage 16, 754–764, doi: 10.1006/nimg.2002.1092 (2002). 12169259

[b48] Kresse G. & Furthmüller J. Efficient iterative schemes for ab initio total-energy calculations using a plane-wave basis set. Physical Review B 54, 11169–11186 (1996). 10.1103/physrevb.54.11169 9984901

[b49] Kresse G. & Hafner J. *Ab initio* molecular-dynamics simulation of the liquid-metal amorphous-semiconductor transition in germanium. Physical Review B 49, 14251–14269 (1994). 10.1103/physrevb.49.14251 10010505

[b50] Kresse G. & Hafner J. Ab initio molecular dynamics for liquid metals. Physical Review B 47, 558–561 (1993). 10.1103/physrevb.47.558 10004490

[b51] Kresse G. & Furthmüller J. Efficiency of ab-initio total energy calculations for metals and semiconductors using a plane-wave basis set. Computational Materials Science 6, 15–50, doi: 10.1016/0927-0256(96)00008-0 (1996).

[b52] Perdew J. P., Ruzsinszky A., Csonka G. I., Constantin L. A. & Sun J. Workhorse Semilocal Density Functional for Condensed Matter Physics and Quantum Chemistry. Physical Review Letters 103, 026403 (2009). 19659225 10.1103/PhysRevLett.103.026403

[b53] Sun J. . Self-consistent meta-generalized gradient approximation within the projector-augmented-wave method. Physical Review B 84, 035117 (2011).

[b54] Corso A. D. & Conte A. M. Spin-orbit coupling with ultrasoft pseudopotentials: Application to Au and Pt. Physical Review B 71, 115106 (2005).

[b55] Christensen N. E. & Seraphin B. O. Relativistic Band Calculation and the Optical Properties of Gold. Physical Review B 4, 3321–3344 (1971).

[b56] Rangel T. . Band structure of gold from many-body perturbation theory. Physical Review B 86, 125125 (2012).

[b57] Gray D. E. American Institute of Physics handbook(McGraw-Hill 1963).

[b58] Neighbours J. R. & Alers G. A. Elastic Constants of Silver and Gold. Physical Review 111, 707–712 (1958).

[b59] Varvenne C., Bruneval F. & Marinica M.-C. & Clouet, E. Point defect modeling in materials: Coupling *ab initio* and elasticity approaches. Physical Review B 88, 134102 (2013).

