# Supplementary material for: 3D lattice distortions and defect structures in ion-implanted nano-crystals
Source: Sci Rep. 2017 Apr 6;7:45993. doi: 10.1038/srep45993 (PMC5382701; doi:10.1038/srep45993)
Supplement: Supplementary Information [file srep45993-s1.pdf]

Supplementary Information: 3D lattice distortions and defect structures in ion-implanted nano-crystals

Felix Hofmann *[1], Edmund Tarleton[2], Ross J. Harder[3], Nicholas W. Phillips[4,5], Pui-Wai Ma[6], Jesse N. Clark[7], Ian K. Robinson[8], Brian Abbey[4], Wenjun Liu[3], and Christian E. Beck[2]

[1]Department of Engineering Science, University of Oxford, Parks Road, Oxford, OX1 3PJ, UK
[2]Department of Materials, University of Oxford, Parks Road, Oxford, OX1 3PH, UK
[3]Advanced Photon Source, Argonne National Laboratory, Argonne, Illinois 60439, USA
[4]ARC Centre of Advanced Molecular Imaging, Department of Chemistry and Physics, La Trobe Institute for Molecular Science, La Trobe University, Victoria 3086, Australia
[5]CSIRO Manufacturing Flagship, Parkville 3052, Australia
[6]Culham Centre for Fusion Energy, Culham Science Centre, Abingdon, Oxfordshire, OX14 3DB, UK
[7]Stanford PULSE Institute, SLAC National Accelerator Laboratory, Menlo Park, CA 94025, USA
[8]Brookhaven National Laboratory, 734 Brookhaven Avenue, Upton, NY, 11973, USA

1 SI Notes

1.1 Analysis of defects in crystal C

Several further defects can be identified in crystal C, in addition to the dislocation examined in the main text. SI Fig. S6 shows a 3D rendering of crystal C with the $\mathbf{q}_{hkl}$ vector directions of the six measured reflections superimposed. Amplitude and phase of the complex electron density reconstructed from each reflection are shown on two slices though the crystal. In slice I two defects, (a) and (b) can be identified. Defect (a), is described in the main text. In slice II defects (c) and (d) are visible. The phase jumps associated with each defect, observed in the phases of the measured reflections, are listed SI Table S2). As discussed in the main text the magnitude of the expected phase jump, $\Delta\psi_{hkl}$, due to a dislocation with burgers vector $\mathbf{b}$, observed in a reflection with scattering vector $\mathbf{q}_{hkl}$, is $\Delta\psi_{hkl} = \mathbf{b} \cdot \mathbf{q}_{hkl}$.

The scattering vectors associated with the six reflections measured from crystal C are: $\mathbf{q}_{[-111]} = \frac{2\pi}{a}$[-111], $\mathbf{q}_{[1-11]} = \frac{2\pi}{a}$[1-11], $\mathbf{q}_{[11-1]} = \frac{2\pi}{a}$[11-1], $\mathbf{q}_{[200]} = \frac{2\pi}{a}$[200], $\mathbf{q}_{[020]} = \frac{2\pi}{a}$[020] and $\mathbf{q}_{[002]} = \frac{2\pi}{a}$[002]. Hence the Burgers vectors listed in the final column of SI Tab. S2) can be assigned to defects (a), (c) and (d). All defects can be identified as stair-rod dislocations with $\mathbf{b} = \frac{a}{3}$ <110>. For defect (b) the analysis is less conclusive, with inconsistent phase jumps observed in the different reflections. This is most likely due to close proximity of defect (b) to a further defect, seen as a reduction in (200) amplitude close to the dip in amplitude from defect (b) (see slice I in SI Fig. S6).

*felix.hofmann@eng.ox.ac.uk

2 SI Tables

	Number of atoms	Supercell size (cubic unit cell)	k-points	Formation energy (eV)	Relaxation volume (atomic volume)
Vacancy	107	3x3x3	4x4x4	0.94	-0.38
100 dumbbell	145	3x3x4	4x4x3	3.93	2.02
Octahedral	145	3x3x4	4x4x3	4.10	2.06
110 crowdion	193	3x4x4	4x3x3	3.94	2.00
110 dumbbell	193	3x4x4	4x3x3	3.95	2.00
Substitution Ga	108	3x3x3	4x4x4		0.057

Supplementary Table S1: The formation energy and relaxation volume of mono-vacancy, 100 dumbbell, octahedral site, 110 crowdion and 110 dumbbell self-interstitial configurations, and the relaxation volume of a substitutional Ga atom in Au.

Defect	Phase jump in different reflections (rad)							Burgers vector
		(-111)	(1-11)	(11-1)	(200)	(020)	(002)	
a	measured	0	-4.1	4.2	0	4.2	-4.3	$\frac{a}{3}$[01-1]
	calculated	0	$-\frac{4}{3}\pi$	$\frac{4}{3}\pi$	0	$\frac{4}{3}\pi$	$-\frac{4}{3}\pi$	
b	measured	-3.4	3.4	0	3.4	-2	0	
	calculated							
c	measured	-4.3	4.6	0	4.6	-4.5	0	$\frac{a}{3}$[1-10]
	calculated	$-\frac{4}{3}\pi$	$\frac{4}{3}\pi$	0	$\frac{4}{3}\pi$	$-\frac{4}{3}\pi$	0	
d	measured	-4.2	0	4.3	4.1	0	-4.4	$\frac{a}{3}$[10-1]
	calculated	$-\frac{4}{3}\pi$	0	$\frac{4}{3}\pi$	$\frac{4}{3}\pi$	0	$-\frac{4}{3}\pi$	

Supplementary Table S2: Summary of four defects analysed in sample C, shown in SI Fig. S6. Column 1 shows the defect designator. Columns 2 - 8 show the measured phase jump and calculated phase jump for each defect in all six reflections. The last column list the burgers vector likely to be associated with each defect.

3 SI Figures

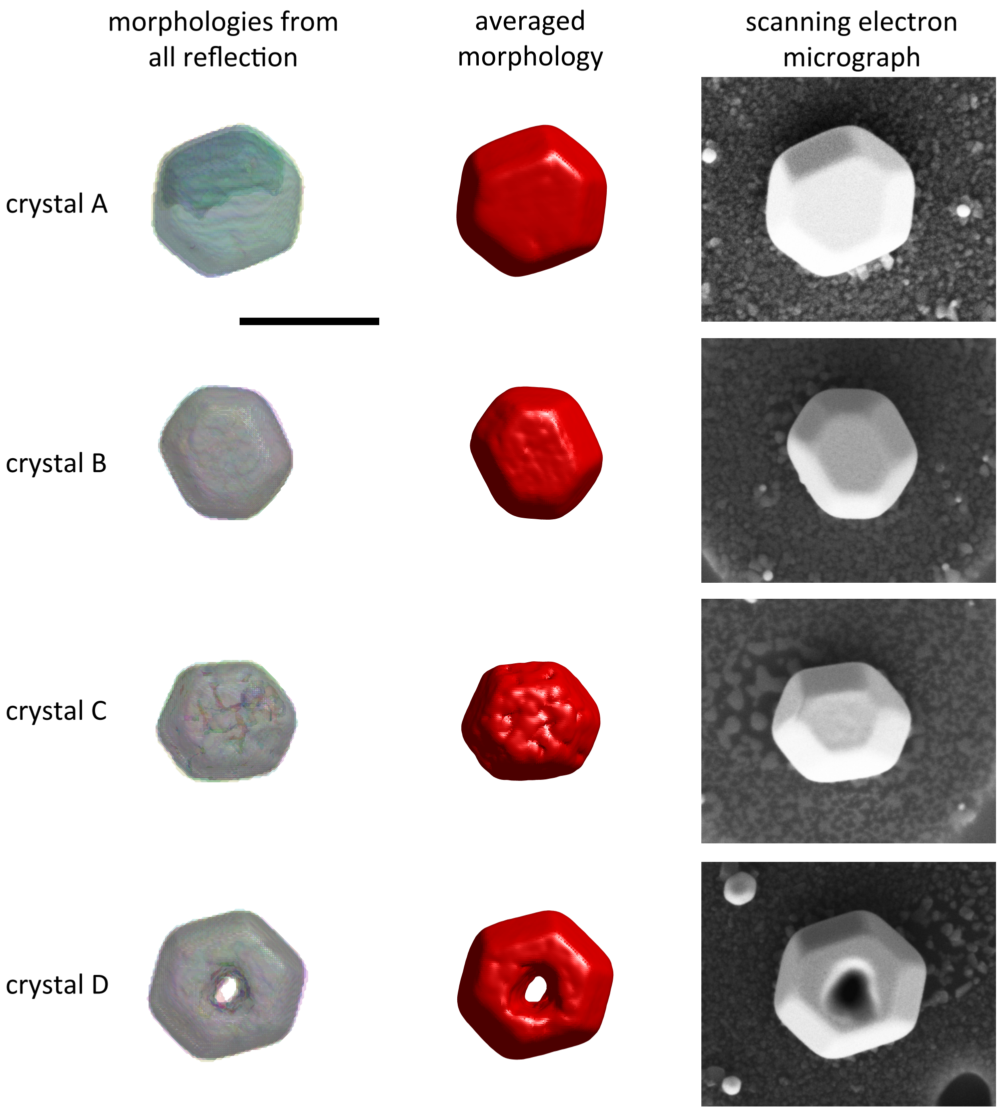

Supplementary Figure S1: Comparison of sample morphologies recovered using BCDI and scanning electron micrographs of crystals A, B, C and D. The first column shows, for each crystal, a superposition of the morphologies recovered from different crystal reflections using BCDI. The second column shows the average morphology of each crystal determined by averaging over the morphologies found from different lattice reflections of that crystal. Scanning electron microscopy images of all four crystals are shown in the third column. All plots are shown at the same magnification and using the same viewpoint. The scalebar corresponds to 1 μm.

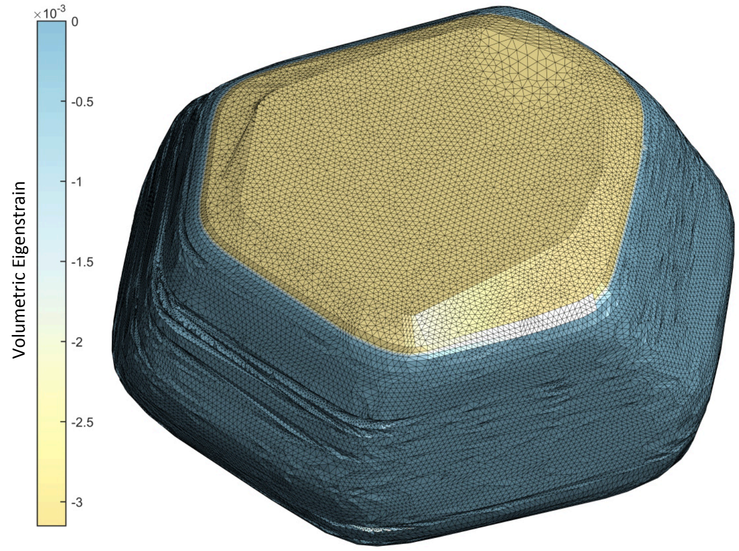

Supplementary Figure S2: 3D visualization of crystal A finite element model. The model mesh is shown and the model is colored according to the imposed volumetric strain loading, ϵ_v, showing the lattice contraction applied to the implanted surface layer.

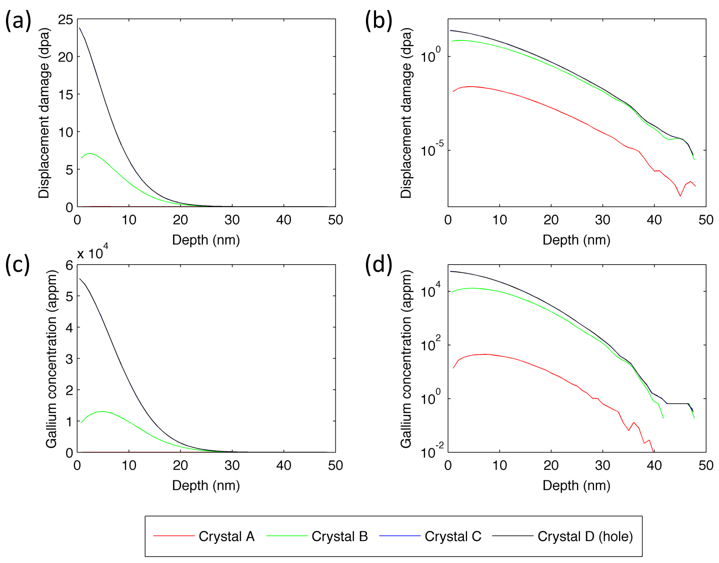

Supplementary Figure S3: Displacement damage and gallium implantation profiles calculated using SRIM. (a) Displacement damage, in displacements per atom (dpa), plotted on a linear scale. (b) Displacement damage plotted on a logarithmic scale. (c) Implanted gallium, in atomic parts per million (appm), plotted on a linear scale. (d) Implanted gallium plotted on a logarithmic scale.

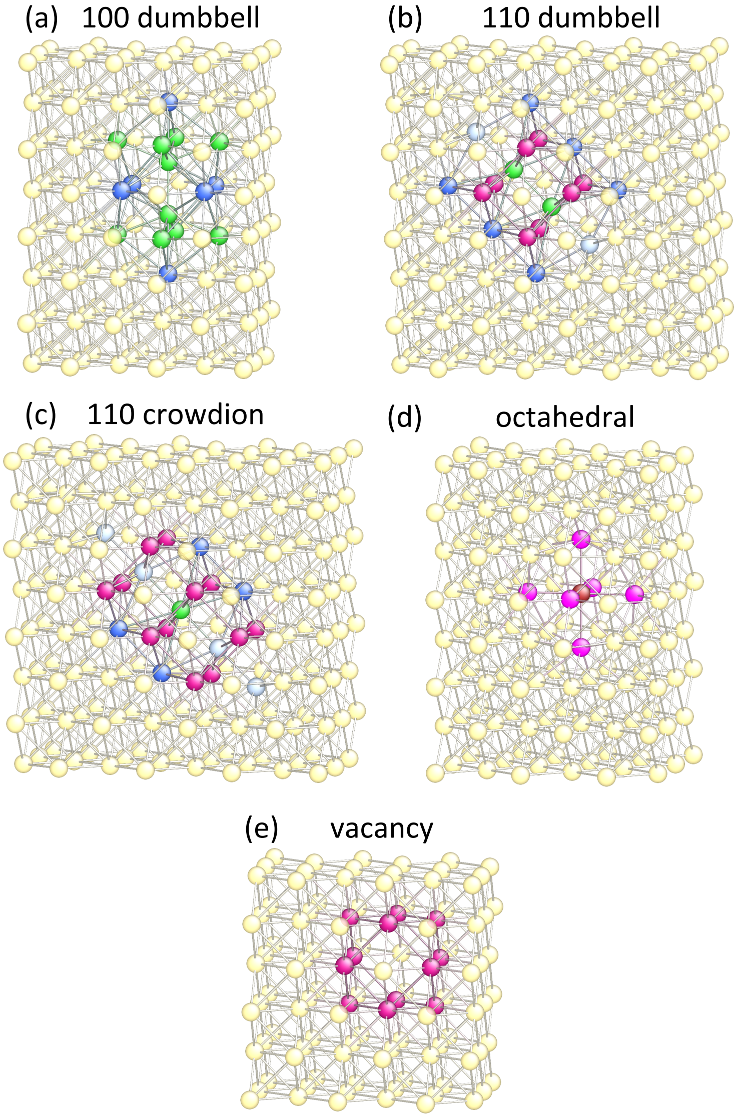

Supplementary Figure S4: Visualisation of the supercells used for DFT calculations of point defect properties in gold. Interstitial configurations: (a) 100 dumbbell, (b) 100 dumbbell, (c) 110 crowdion and (d) octahedral site. The configuration of a vacancy is shown in (e). Atoms are color coded by coordination number: brown (6), light blue (8), magenta (9), green (10), yellow (12) and dark blue (13).

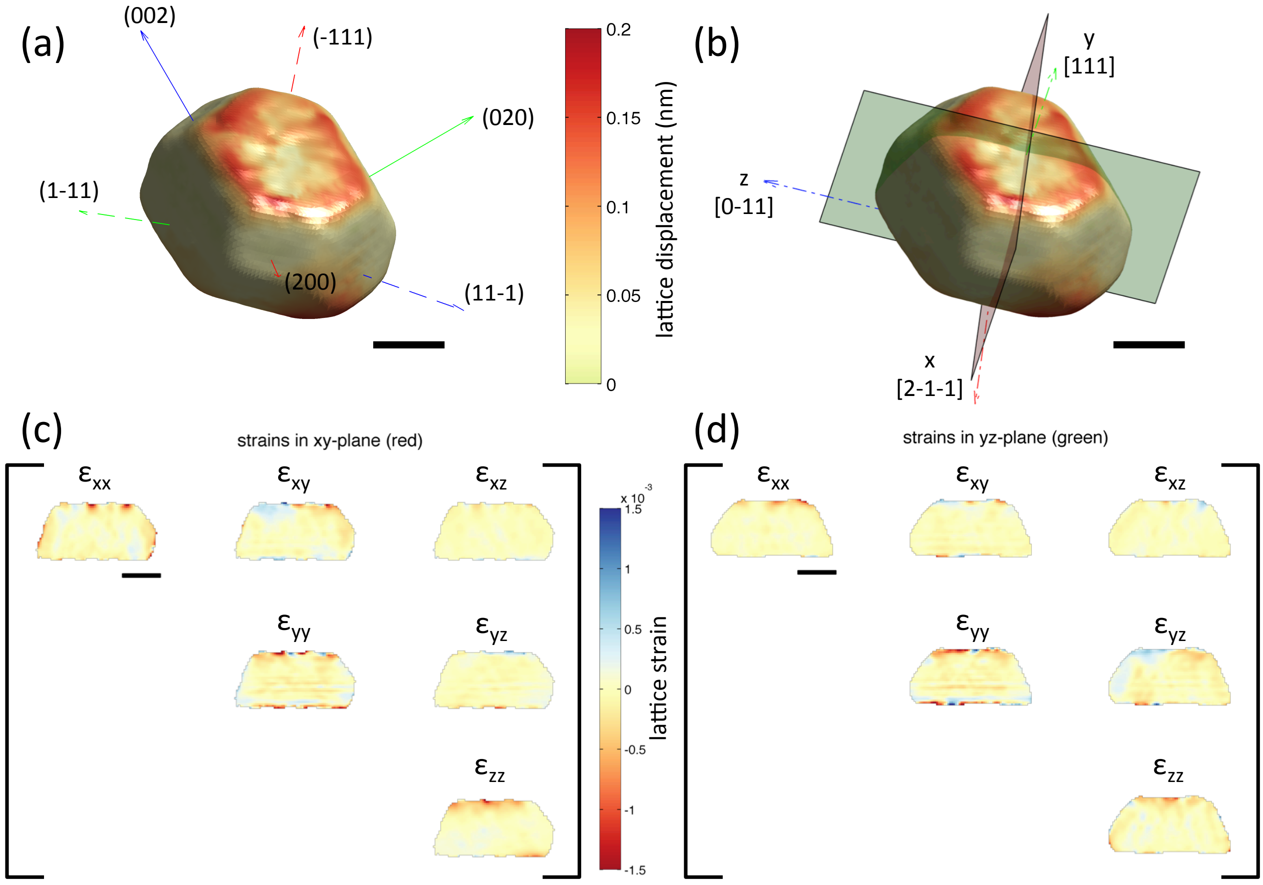

Supplementary Figure S5: Full lattice strain tensor in crystal B after Ga-ion implantation. (a) 3D rendering of crystal B coloured according to the magnitude of the lattice displacement field. Superimposed are the q vectors of the 6 crystal reflections that were measured. (b) Crystal coordinate system used for plotting of lattice strains. (c) Maps of the six independent lattice strain tensor components on an xy section through crystal B (red plane in (b)). (d) Maps of 6 strain tensor components on a yz section though crystal B (green plane in (b)). Scale bars are 300 nm in length.

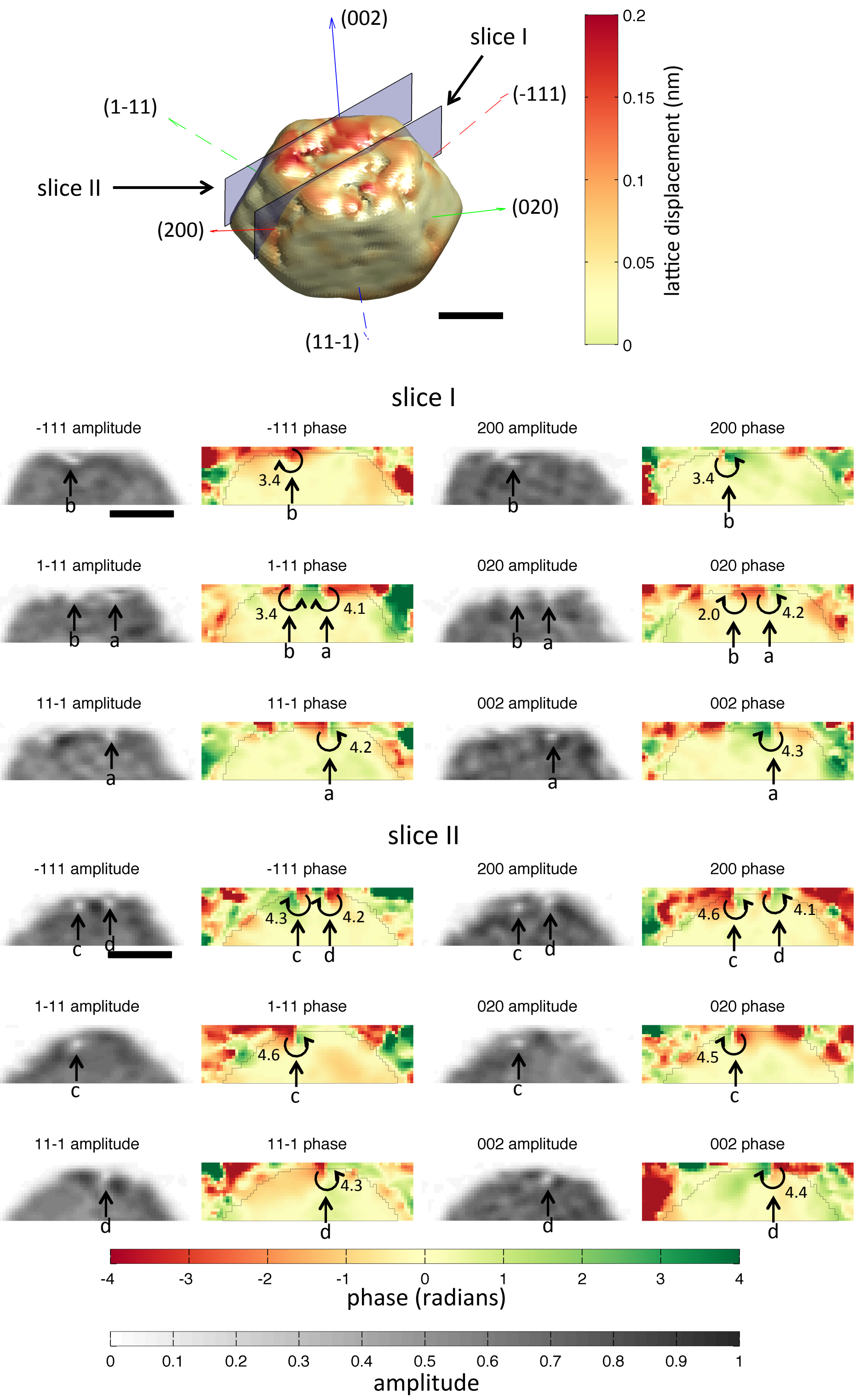

Supplementary Figure S6: Lattice defects in crystal C. 3D rendering of crystal C, colored according to lattice displacement. Superimposed are the six reflections for which BCDI measurements were carried out. Also shown are two slices for which the amplitude and phase of the complex electron density, recovered from all six lattice reflections, are shown. In both slice I and slice II amplitude and phase features due to defects are visible and four distinct defects have been labelled (a) to (d). The direction of the phase jump associated with specific defects is marked by a circular arrow in the phase maps and its approximate value is noted in radians. A summary of this data is provided in SI Table S2. Scale bars are 300 nm in length.

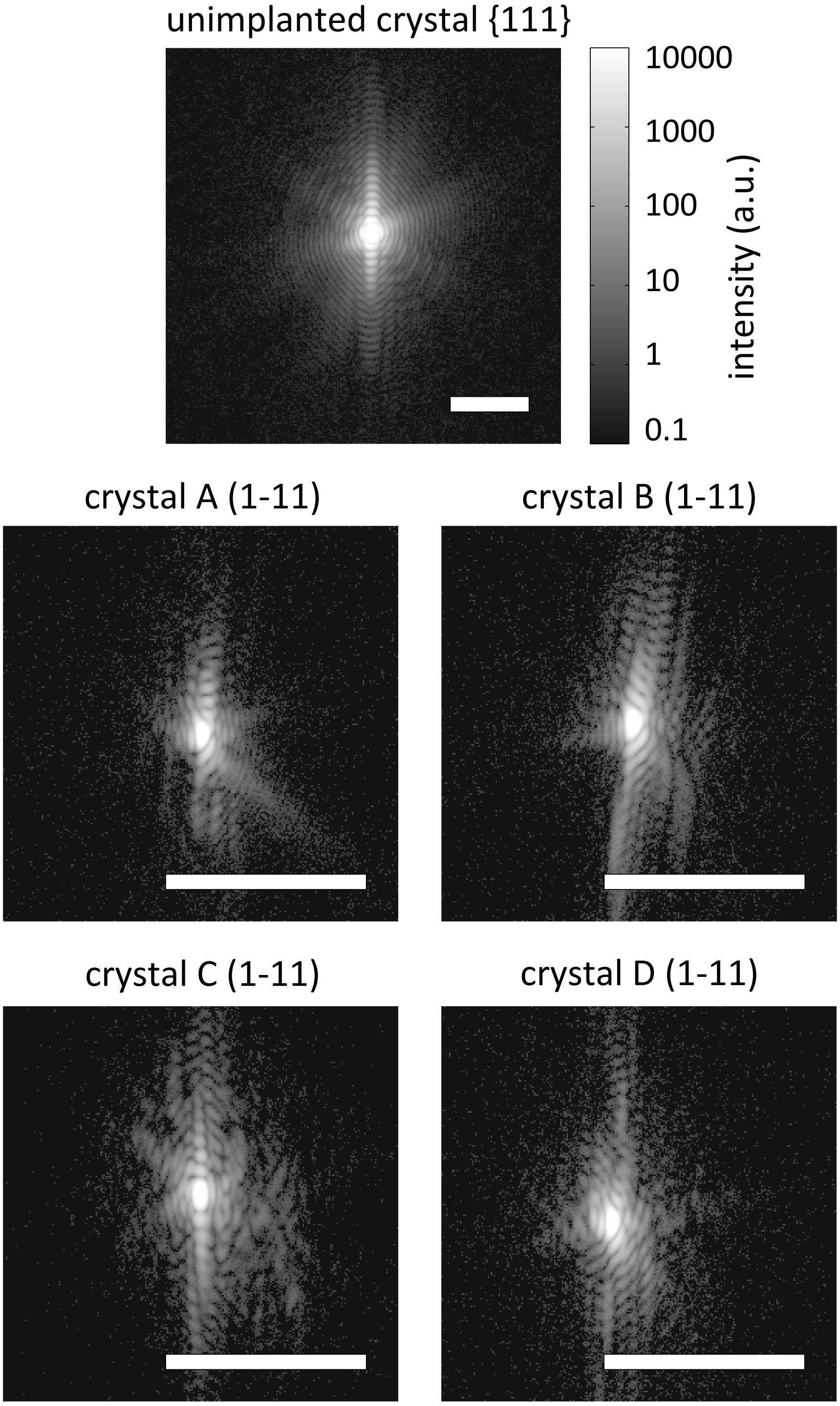

Supplementary Figure S7: Coherent {111} diffraction patterns from the unimplanted reference crystal and crystals A, B, C and D. The scale bars correspond to a $\mathbf{q} = 0.2$ nm^{-1}.
